# Metformin: A Novel Weapon Against Inflammation

**DOI:** 10.3389/fphar.2021.622262

**Published:** 2021-01-29

**Authors:** Bo Bai, Haibo Chen

**Affiliations:** Department of Cardiology, Shenzhen Second People’s Hospital, The First Affiliated Hospital of Shenzhen University Health Science Center, Shenzhen, China

**Keywords:** metformin, inflammatin, clinical utilization, debate, chronic disease

## Abstract

It has become widely accepted that inflammation is a driving force behind a variety of chronic diseases, such as cardiovascular disease, diabetes, kidney disease, cancer, neurodegenerative disorders, etc. However, the existing nonsteroidal anti-inflammatory drugs show a limited utility in clinical patients. Therefore, the novel agents with different inflammation-inhibitory mechanisms are worth pursuing. Metformin, a synthetic derivative of guanidine, has a history of more than 50 years of clinical experience in treating patients with type 2 diabetes. Intense research efforts have been dedicated to proving metformin’s inflammation-inhibitory effects in cells, animal models, patient records, and randomized clinical trials. The emerging evidence also indicates its therapeutic potential in clinical domains other than type 2 diabetes. Herein, this article appraises current pre-clinical and clinical findings, emphasizing metformin’s anti-inflammatory properties under individual pathophysiological scenarios. In summary, the anti-inflammatory effects of metformin are evident in pre-clinical models. By comparison, there are still clinical perplexities to be addressed in repurposing metformin to inflammation-driven chronic diseases. Future randomized controlled trials, incorporating better stratification/targeting, would establish metformin’s utility in this clinical setting.

## Introduction

The hypoglycemic activity of metformin, a synthetic derivative of guanidine, was first described in 1968 (Clarke and Duncan, 1968). After that, it is prevalently used as a first-line treatment for type 2 diabetes (T2D) (Stumvoll et al., 1995; Knowler et al., 2002). The glucose-lowering effect of metformin is primarily attributable to its capability to regulate energy metabolism, including inhibition of hepatic gluconeogenesis, reduction of glucose absorption, and elevation of glucose utilization in peripheral tissues (Foretz et al., 2014). The activation of the cellular energy sensor [AMP-activated protein kinase (AMPK)] is the most extensively studied mechanisms of metformin in hepatocytes. By blocking the complex-I of the respiratory chain in mitochondria, metformin suppresses adenosine triphosphate (ATP) production, increases cytoplasmic adenosine monophosphate (AMP): ATP ratios, leading to activation of AMPK. Metformin is also found to activate AMPK by increasing the net phosphorylation of the catalytic *α* subunit of AMPK at threonine 172 (Zhou et al., 2001; He and Wondisford, 2015). A recent study unraveled that metformin might activate AMPK by a mechanism involving the lysosome (Zhang et al., 2016). Subsequently, the activated AMPK phosphorylates the acetyl-CoA carboxylase (ACC), by which metformin can regulate lipid homeostasis and enhance insulin sensitivity (Fullerton et al., 2013).

Beyond its consolidated role in T2D management, the pleiotropic actions of metformin have been extensively documented. Metformin can treat cardiovascular diseases by mechanisms distinct from its metabolic activities (Soraya et al., 2014; Liu et al., 2017; Dziubak et al., 2018). Metformin exerts nephroprotective effects in diabetic patients (Kawanami et al., 2020) and interferes with key immunopathological molecules involved in tumor progression (Ma et al., 2020). These findings, together with one particular breakthrough in metformin-induced longevity in microbes and mice (Cabreiro et al., 2013; Martin-Montalvo et al., 2013; Chen et al., 2017), provide the possibility of boosting its therapeutic potential in treating aging and age-related diseases (Storelli et al., 2013). Of note, emerging *in vitro* and *in vivo* evidence suggests that metformin can exert potent inflammation-inhibitory effects, irrespective of its capability of glucose control (Saisho, 2015; Bharath et al., 2020). Most recently, it is of great interest to find that metformin is able to dampen cytokine storms in patients who are infected with coronavirus disease 2019 (COVID-19). The use of metformin is significantly associated with reduced circulating levels of inflammatory markers (Cheng et al., 2020) and decreased in-hospital mortality (Hariyanto and Kurniawan, 2020; Kow and Hasan, 2020; Luo et al., 2020). It has become widely accepted that inflammation is a driving force behind various chronic diseases, including heart failure, atherosclerosis, diabetes, obesity, neurodegenerative disease, cancer, etc. The reduction in lifetime exposure to inflammation has contributed to the historical decline in old-age mortality (Finch and Crimmins, 2004; Couzin-Frankel, 2010). Herein, this article will appraise current pre-clinical and clinical findings with special emphasis on metformin’s anti-inflammatory properties under individual pathophysiological scenarios.

## Cardiovascular Diseases

Metformin can protect against cardiovascular diseases (CVD) associated with inflammatory stress, such as endothelial dysfunction (Tian et al., 2019), myocardial infarction (Soraya et al., 2014), acute myocarditis (Liu et al., 2017), and chronic heart failure (Dziubak et al., 2018). In human umbilical vein endothelial cells (HUVECs), metformin activates AMPK and subsequently enhances phosphorylation of histone deacetylase 5 (HDAC5) at serine 498, which leads to up-regulation of transcription factor Kruppel-like factor 2 (KLF2). Thereby, metformin eliminates lipopolysaccharide (LPS) or tumor necrosis factor *a* (TNFα)-induced vascular cell adhesion molecule 1 (VCAM1) expression (Tian et al., 2019). Metformin can inhibit TNFα-induced NF-kappaB (NF-κB) activation and transcriptionally suppress expression of E-selectin, intercellular adhesion molecule-1 (ICAM-1), and monocyte chemoattractant protein-1 (MCP-1) in HUVECs (Hattori et al., 2006). Metformin dampens high glucose-induced mitochondrial fragmentation by down-regulating dynamin-related protein-1 (Drp-1) in HUVECs. Consequently, metformin abrogates the increase of ICAM-1 and VCAM-1 in the aortic endothelium of diabetic ApoE deficient mice. However, these beneficial effects of metformin are abolished upon AMPK α2 deficiency (Wang et al., 2017). In primary cultured rat vascular smooth muscle cells (VSMCs), metformin activates AMPK-induced phosphatase and tensin homolog (PTEN) expression. Through the AMPK-PTEN pathway, metformin negatively regulates cyclooxygenase (COX)-2 and inducible nitric oxide synthase (iNOS) expression in VSMCs treated with TNFα (Kim and Choi, 2012). Apart from AMPK activation, metformin strikingly down-regulates microRNA-21 (miR-21) in a time- and dose-dependent manner in HUVECs. The miR-21 directly targets the 3′-UTR of PTEN and negatively regulates PTEN expression (Luo et al., 2017). The fatty acid (palmitic acid) stimulates an inflammatory response, associated with a decreased level of miR-155 that can negatively regulate mRNA levels of MCP-1 and ICAM-1 in human aortic endothelial cells. Metformin treatment antagonizes endothelial inflammation by up-regulation of miR-155 (Gou et al., 2020). Diabetic Goto-Kakizaki (GK) rats with metformin treatment have decreased superoxide production and reduced advanced glycation end-products accumulation in the vasculature. Moreover, levels of the chemokines MCP-1, one of the earliest molecular markers of vascular inflammation in atherogenesis, are significantly attenuated in the rat aortae (Sena et al., 2011). In other ways, metformin antagonizes vascular inflammation by inhibiting the monocyte-to-macrophage differentiation that critically accentuates atherosclerosis through promoting an inflammatory environment within the vessel wall. The phorbol myristate acetate‐induced THP-1 monocyte differentiation is blunted by metformin, accompanied by reduced cellular IL-1β, TNF-α, and MCP-1 levels. The phosphorylation of signal transducer and activator of transcription 3 (STAT3) plays a critical role in mediating monocyte-to-macrophage differentiation and inflammation, but all of which are dose-dependently inhibited by metformin (Vasamsetti et al., 2015). Moreover, metformin can reduce cytokine secretion (IL-12p40 and IL-6) from mouse bone marrow-derived macrophages *in vitro* (Cameron et al., 2016). Male Wistar rats develop acute myocardial infarction after subcutaneous injection with isoproterenol. Pre-treatment with metformin protects left ventricular dysfunction and enhances phosphorylation of AMPKα, coincided with markedly decreased gene expression of toll-like receptor4 (TLR4), myeloid differentiation protein 88 (MyD88), TNF-α, and IL-6 in the post-infarct heart tissues (Soraya et al., 2014). Male C57BL/6 mice challenged with ischemia/reperfusion (I/R) injury via left coronary artery ligation exhibit myocardial apoptosis, inflammation, and collagen deposition. Treatment with metformin significantly attenuates I/R induced pathological changes and cellular apoptosis in mouse hearts. The inhibition of autophagosome formation and restoration of the impaired autophagosome processing contributes to metformin’s cardioprotective effect, dependent on the Akt signaling pathway (Huang et al., 2020). BALB/c mice exposed to LPS challenge have an elevation of creatinine kinase-myocardial band (CK-MB) and brain natriuretic peptide (BNP) in plasma. The pro-inflammatory cytokine levels, such as TNF-α, IL-1β, IL-6, myeloperoxidase (MPO), are augmented in mouse myocardium. Metformin attenuates endotoxin-induced acute myocarditis via activating AMPK-dependent anti-inflammatory mechanism (Liu et al., 2017). Male C57BL/6J develop lung injury and cardiac dysfunction after inhaling fine particulate matter (PM2.5) airborne pollution. Administration of metformin effectively reduces systemic and pulmonary inflammation, preserves left ventricular ejection fraction, suppresses pulmonary and myocardial fibrosis, and eliminates oxidative stress. Metformin restrains cardiac superoxide production by enhancing protein levels of superoxide dismutase 2 (SOD2), peroxiredoxin (PRDX) 3 and 5, and thioredoxin reductase 2 (TRXR2). Of note, metformin also protects PM2.5-induced lung injury and cardiac dysfunction in AMPKα2 deficient mice, suggesting a pathway that appears independent of AMPK (Gao et al., 2020). Reactive dicarbonyls stimulate the production of advanced glycation end-products, increase oxidative stress and inflammation. Hereditary hyper-triglyceridemic rats have increased concentrations of reactive dicarbonyls in the myocardium and kidney cortex. Metformin treatment significantly reduces reactive dicarbonyls, along with elevated glutathione (GSH) and glyoxalase-1 mRNA expression in mouse heart tissues (Malinska et al., 2018). Metformin also protects against inflammation and dicarbonyl stress in the left ventricles of spontaneously hypertensive rats that have a transgenic expression of human C-reactive protein (CRP). The treatment results in decreased circulating inflammatory markers in rats, including IL-6, TNFα, and MCP-1 (Malinska et al., 2016). The administration of metformin to the adult male albino rats that have been exposed to the whole-body gamma radiation ameliorates the elevation of cardiac injury biomarkers, including lactate dehydrogenase (LDH) and CK-MB. In addition, heart catalase and superoxide production, as well as NF-κB, IL-6 and TNF-α levels, are markedly decreased in metformin-treated rats, which thus may hold a promise for the implication of metformin as an adjunct to radiotherapy (Karam and Radwan, 2019).

Clinically, three randomized controlled trials are providing the most crucial evidence of the cardiovascular protective effects of metformin, including the UK Prospective Diabetes Study (UKPDS) [UK Prospective Diabetes Study (UKPDS) group, 1998] and two prospective clinical trials [NCT00375388 (Kooy et al., 2009) and NCT00513630 (Hong et al., 2013)]. The UKPDS Group investigates the effect of intensive blood-glucose control with metformin on complications in overweight T2D patients. The group reports that 342 obese diabetic patients treated with metformin have risk reductions for any diabetes-related endpoint (−32%), diabetes-related death (−42%), and all-cause mortality (−36%) [UK Prospective Diabetes Study (UKPDS) group, 1998]. The post-trial follow-up for ten years further demonstrates that patients with metformin treatment have a significantly reduced risk for any diabetes-related endpoint (−21%), myocardial infarction (−33%), and death from any cause (−27%) (Holman et al., 2008). The second clinical trial (NCT00375388) observes the long-term effects of metformin on the microvascular and macrovascular disease in T2D patients. The conclusion is that metformin, added to insulin in diabetic patients, fails to improve the primary endpoint (an aggregate of microvascular and macrovascular morbidity and mortality). However, it does reduce the risk of macrovascular disease after a follow-up period of 4.3 years (Kooy et al., 2009). The third clinical trial (NCT00513630) compares the long-term effects of glipizide and metformin on the major cardiovascular events in T2D patients with a history of coronary artery disease. The results demonstrate that metformin treatment for three years substantially reduces major cardiovascular events in patients after a median follow-up of five years, compared with glipizide (Hong et al., 2013). Moreover, a recent study reports that, in a cohort of 1,213 hospitalized patients with COVID-19 and pre-existing T2D, metformin use is significantly associated with reduced heart failure and decreased circulating inflammatory markers, such as CRP, IL-6, IL-2, and TNF-α. Metformin users also have a low neutrophil to lymphocyte ratio (NLR) in the blood (Cheng et al., 2020). Metformin treatment shows the potential to reduce cardiac risk in postpartum women with gestational diabetes (GDM). Postpartum women who receive metformin have an apparent reduction of circulating oxidized low-density lipoprotein cholesterol (LDL) (Viteri et al., 2017). Despite encouraging data reported in diabetic CVD patients, metformin’s benefits to non-diabetic patients remain mixed so far. In non-diabetic patients with heart failure, metformin therapy is associated with a pronounced reduction of circulating cytokines, such as IL-2, IL-4, and C-X-C motif chemokines ligand 12 (CXCL12) (Cameron et al., 2016). In non-diabetic women with chest pain and a prior history of normal coronary angiography, metformin recipients show improved vascular function and a decreased incidence of myocardial ischemia (Jadhav et al., 2006). By contrast, the Glycometabolic Intervention as Adjunct to Primary Percutaneous Coronary Intervention in ST-Segment Elevation Myocardial Infarction (GIPS) III clinical trial demonstrates that, among 379 non-diabetic patients with ST-elevation myocardial infarction, four months of metformin treatment only results in a modest improvement of the cardiovascular risk profile. Metformin treatment reduces glycated hemoglobin, total cholesterol, LDL, and body mass index of patients. However, levels of fasting glucose, insulin, high-density lipoprotein cholesterol (HDL), and blood pressure are similar between the metformin and placebo groups (Lexis et al., 2015). GIPS III clinical trial also determines the effect of metformin on left ventricular function after acute myocardial infarction in patients without diabetes. The study concludes that, compared with placebo, the use of metformin does not result in improved left ventricular ejection fraction or NT-proBNP level (Lexis et al., 2014). The two-years follow-up results further demonstrate that the incidence of major adverse cardiac events is comparable between the metformin and placebo groups (Hartman et al., 2017). In non-diabetic cohorts with coronary heart disease and large waist circumferences who have received statins, metformin treatment does not affect mean distal carotid intima-media thickness. It has little or no effect on several surrogate markers of CVD, such as carotid plaque score, hemoglobinA1c (HbA1c), total cholesterol, HDL, non-HDL-cholesterol (total cholesterol value minus HDL), triglycerides, and high sensitivity CRP (Preiss et al., 2014).

## Kidney Diseases

Persistent systemic inflammation is highly prevalent in patients with chronic kidney disease (CKD). The acute or chronic-phase of inflammation frequently accompanies the declining renal function (Krane and Wanner, 2011). The pre-clinical studies have provided compelling evidence that metformin protects renal injuries by abating inflammation insulted by different stimuli. *In vitro* study unravels that hyperglycemia impairs glucagon-like peptide-1 receptor (GLP-1R) expression in HBZY-1 rat mesangial cell line, paralleled with NF-κB activation and increased MCP-1 protein levels. Metformin can restore GLP-1R mRNA expression and decrease high glucose-evoked inflammation in HBZY-1 cells (Kang et al., 2019). Metformin inhibits TGF-β1-stimulated MCP-1, Type IV collagen, and fibronectin expression in human proximal tubular cells (HK2 cells). The underlying mechanism is that metformin reduces the expression of SMAD family member 3 (Smad3), phosphorylation of ERK1/2 and P38 (Yi et al., 2018). The pregnant C57BL/6 mice fed with a high-fat diet develop GDM. Pre-treatment with metformin significantly decreases urine microalbumin levels and serum β2-microglobulin in GDM mice during late pregnancy. Furthermore, GDM mice following metformin treatment have reduced serum levels of IL-6, TNF-α, and low phosphorylation of MAPK1/3, MAPK14, and MAPK8 in the kidneys (Liu et al., 2019). In addition, metformin treatment can protect diabetic nephropathy by improving glycoxidation, together with inhibition of fibrosis and inflammation in the kidneys of diabetic GK rats fed with an atherogenic diet. The renal tissue levels of IL-1β, TGF-β1 are reduced by metformin treatment (Louro et al., 2011). Metformin exerts beneficial effects on obesity-induced renal injury in young C57BL/6 mice fed with a high-fat diet. Metformin treatment rescues renal AMPK activity and fatty acid oxidation. It substantially decreases glomerular mesangial matrix expansion and macrophage infiltration in mouse kidney tissues (Kim et al., 2013). In C57BL/6 mice with folic acid-induced nephropathy, metformin is able to reduce urinary albumin excretion and prevent renal inflammatory responses and tubulointerstitial fibrosis. The renal levels of TGF-β1, Type IV collagen, and fibronectin are substantially repressed by metformin treatment (Yi et al., 2018). The renal proximal tubule-specific Tsc1 gene-knockout (Tsc1 ptKO) mice develop aberrantly enlarged kidneys primarily that is due to hypertrophy and proliferation of proximal tubule cells, concurrent with interstitial inflammation and fibrosis. However, metformin treatment increases renal AMPK phosphorylation but decreases the Akt phosphorylation. These signaling modulations effectively attenuate histopathological changes and functional decline in kidney tissues of Tsc1 ptKO mice (Fang et al., 2020). Metformin preconditioning prevents renal tubular epithelial cell apoptosis and inflammation in kidneys of Sprague-Dawley (SD) rats challenged with ischemia and renal arteriovenous perfusion. Histologically, there are fewer renal tubular necrotizing changes in the metformin group than that in the ischemia group. The phosphorylation of AMPK is enhanced, but caspase 3 and COX-2 levels are decreased by metformin treatment (Wang et al., 2015). Administration with metformin also antagonizes the tubule dilation and interstitial inflammation caused by ureteral obstruction. In a mouse model of unilateral ureteral obstruction (UUO), metformin reduces the expression of extracellular matrix proteins (collagen and fibronectin) and profibrotic TGF‐β1 in obstructed kidneys, dependent on AMPK activation. UUO‐triggered interstitial fibroblast activation is ameliorated by metformin, which is demonstrated by the reduction of renal *a*-smooth muscle actin (α-SMA) (Cavaglieri et al., 2015). Interestingly, another study reports that in AMPK β1 deficient mice challenged with UUO, metformin still protects renal injury histologically and functionally, suggesting the protective effects of metformin are not dependent specifically on activation of AMPK in mouse kidney tissue (Christensen et al., 2016). The use of cyclosporine A (CsA) as an immunosuppressive agent is often limited owing to its nephrotoxic properties. Administration of metformin and silymarin ameliorates CsA-induced functional damages to kidneys of Wistar albino rats. Significant protection of oxidative stress (increased SOD activity and GSH levels), inflammation (decreased MPO and TNF-α) is observed in kidney tissues of rat following metformin treatment. Normalization of histological changes, as well as COX-2 and iNOS immunoreactivity scores, further strengthens these findings (Vangaveti et al., 2020).

Despite the aforementioned renal protective effects of metformin in cells or animal models, it remains great cautious about metformin’s clinical application in the setting of kidney diseases because of the perceived risk of lactic acidosis (Lebacq and Tirzmalis, 1972; Misbin et al., 1998). The complex interplay between metformin, kidney injury, and lactic acidosis have been comprehensively reviewed by Rhee et al. (Rhee and Kalantar-Zadeh, 2017). By comparison, a few studies recently present data indicating that metformin treatment appears to be still pharmacologically efficacious and safe in patients with renal impairment. However, the dose should be carefully adjusted based on patients’ renal function (Bell et al., 2017; Dissanayake et al., 2017; Lalau et al., 2018). The appropriate daily dosing schedules are suggested as 1,500 mg for patients with severe CKD stage 3A, 1,000 mg for CKD stage 3B, and 500 mg for CKD stage 4. Hyperlactatemia is found to be absent among the CKD stage groups (Lalau et al., 2018). In patients with stage 4 diabetic nephropathy, treatment with four weeks of low-dose metformin (250–1000 mg, once daily) is not associated with adverse safety outcomes and revealed stable pharmacokinetics (Dissanayake et al., 2017). The United States FDA has relaxed its recommendation to allow metformin use in patients with mild to moderate renal impairment [estimated glomerular filtration rate (eGFR): 30–60 mL/min/1.73 m^2^], but metformin use is contraindicated in patients with eGFR values <30 mL/min/1.73 m^2^ (Rhee et al., 2017; Flory et al., 2020).

## Neurodegenerative Diseases

Inflammation in the nervous system (“neuroinflammation”), especially when prolonged, can be particularly injurious. The neuroinflammation contributes substantially to disease pathogenesis across both the peripheral (neuropathic pain, fibromyalgia) and central [e.g., Alzheimer’s disease (AD), Parkinson’s disease (PD), ischemia and traumatic brain injury, multiple sclerosis, motor neuron disease, and depression] nervous systems (Skaper et al., 2018). The male Swiss albino mice injected with LPS have systemic inflammation and profound symptoms of sickness behavior. Pre-treatment with metformin significantly reduces systemic and central inflammatory markers, concurrent with protection against LPS-induced oxidative stress (decreased lipid peroxidation but increased GSH levels) in brain tissues (Mudgal et al., 2019). C57 mice challenged with cecal ligation and puncture (CLP) exhibit septic brain damage. Metformin can increase survival percentage, decrease brain edema, preserve the blood-brain barrier (BBB), and improve cognitive function. By activating PI3K/Akt signaling, metformin reduces the neuronal apoptosis induced by sepsis (Tang et al., 2017). Metformin also protects sepsis-associated encephalopathy in Wistar rats after CLP. Treatment with metformin decreases high mobility group box (HMGB1) levels in rat brains but increases tight junction (TJ) proteins, including claudin-3 and claudin-5 (Ismail Hassan et al., 2020). The experimental traumatic brain injury is induced in SD rats by the weight-dropping procedures. Metformin treatment significantly ameliorates neurological deficit, cerebral edema, and neuronal apoptosis. Mechanism study unravels metformin inhibits nuclear translocation of NF-κB p65 and the phosphorylation of ERK1/2 and p38 MAPK, which leads to decreased pro-inflammatory cytokines production and microglial activation in brains (Tao et al., 2018). Pre-treatment with metformin protects forebrain ischemia injury caused by bilateral common carotid artery occlusion in Wistar rats. The histopathological analysis demonstrates reduced infarct size and leukocyte infiltration, as well as repressed MPO activity in metformin-treated rats (Karimipour et al., 2018). In SD rats subjected to permanent middle cerebral artery occlusion, metformin preconditioning offers neuroprotective effects against ischemic stroke by suppressing brain NF-κB activity and decreasing pro-inflammatory cytokines production in peri-infarct regions. The ischemic injury-associated microgliosis and astrocytosis are also alleviated by metformin (Zhu et al., 2015). The intracerebral hemorrhage model is established in SD rats by infusion of whole blood into the right brain striatum. Metformin treatment protects rats from neurological deficits and preserves the survival of striatal neurons under intracerebral hemorrhage condition. Furthermore, metformin downregulates the levels of apoptotic factors (p-JNK3, p-c-Jun and caspase-3) as well as pro-inflammatory cytokines (IL-1β, IL-4, IL-6, and TNF-α) (Qi et al., 2017). Aging drives substantial molecular to morphological changes in brain. Metformin restores the antioxidant status and improves healthy brain aging in naturally aged and D-galactose‐induced rat models. Metformin augments ferric reducing antioxidant potential, GSH, and Beclin-1 levels, whereas it reduces ROS, protein carbonyl, malondialdehyde, IL-6, and TNF-α in brains of aged rats (Garg et al., 2017). Metformin improves short-term memory and inhibits glial cell activation and neuroinflammation caused by experimental diabetic encephalopathy in C57BL/6 mice injected with streptozotocin. Metformin treatment increases neuronal survival and p-AMPK in hippocampus of diabetic mice. By contrast, metformin significantly represses reactive gliosis, neuronal loss, and NF-kB signaling activation (Oliveira et al., 2016).

PD is a unique neurodegenerative disorder that affects dopamine-producing (“dopaminergic”) neurons predominantly in a specific area of the brain called substantia nigra. Human neuroblastoma SH-SY5Y cells exposed to 1-methyl-4-phenylpyridinium (MPP) exhibit mitochondrial dysfunction and other cellular responses similar to those in the dopaminergic neurons of PD patients. Metformin is able to increase cell viability, correlated with reduced mitochondrial fragmentation and an improvement in the mitochondrial membrane potential (Chanthammachat and Dharmasaroja, 2019). Due to the regulatory role of TNF receptor-associated protein 1 (TRAP1) in mitochondrial energy metabolism control, the homozygous TRAP1 mutation [p. Arg47Ter single nucleotide exchange (R47X)] leads to complete functional protein loss in patients with late-onset PD. Metformin treatment rescues the mitochondrial membrane potential in TRAP1 R47X patients’ fibroblasts, orchestrating with down-regulated phosphorylation of ERK1/2 (Fitzgerald et al., 2017). The murine microglial BV2 cells exposed to LPS show a pro-inflammatory phenotype. However, metformin treatment largely prevents the LPS-induced up-regulation of IL-1β and gene expression of multiple subunits of the NADPH oxidase enzyme, concurrent with decreased ROS generation. Metformin also hinders LPS-induced microglial activation and pro-inflammatory cytokine productions in substantia nigra of Wistar rats, associated with decreased phosphorylation of JNK and p38 (Tayara et al., 2018). The 1-methyl-4-phenyl-1,2,3,6-tetrahydropyridine (MPTP) injection causes neurotoxicity and dopaminergic neurodegeneration in the SN pars compacta of C57 mice. Metformin prevents dopaminergic neuron loss and improves motor behavior in the MPTP mouse model. Metformin induces PGC-1α through activating transcription factor 2 (ATF2)/cAMP response element-binding protein (CREB) signaling, which is critical for the neuroprotective effects of metformin (Kang et al., 2017). One study further reveals that metformin is able to ease MPTP-induced *a*-synuclein phosphorylation and increase the level of a brain-derived neurotrophic factor in the substantia nigra of MPTP-treated C57 mice (Katila et al., 2017). On the contrary, it is reported that, although metformin prevents microglial activation and inflammation in SN pars compacta of MPTP-treated C57 mice, metformin cannot protect MPTP-induced dopaminergic neuron loss. In mesencephalic dopaminergic cells of rat (N27) treated with MPP, metformin can neither preserve cell viability nor reduce ROS generation (Ismaiel et al., 2016).

The clinical evidence that metformin’s effects on PD remain limited. The results obtained from humans do not consistently support the protective effects of metformin on PD. T2D increases PD’s risk by 2.2-fold in humans. Diabetic patients receiving sulfonylureas show an increased risk of PD, but which is avoided by combination with metformin (Wahlqvist et al., 2012). However, in one metformin cohort study that recruits 4,651 patients, metformin exposure exhibits a higher risk of PD than non-users [hazard ratio (HR): 2.27, 95% confidence interval (CI) = 1.68–3.07]. The risk of all-cause dementia is also elevated in metformin users (HR: 1.66, 95% CI = 1.35–2.04) (Kuan et al., 2017). A meta-analysis that includes 285,966 participants further finds no significant effect on the incidence of all the subtypes of neurodegenerative diseases with metformin exposure. Instead, metformin monotherapy is associated with a significantly increased risk of PD incidence (Ping et al., 2020).

AD, the most common neurodegenerative disease worldwide, is characterized by the deposition of amyloid-beta (Aβ) plaques, neurofibrillary tangles, neuronal loss, and neuroinflammation. Unfortunately, pharmacological treatments presently available can merely slow down symptoms but cannot cure the disease. Metformin decreases the apoptosis of Aβ-treated SH-SY5Y neuroblastoma cells by repressing the intracellular concentration of Ca^2+^ and ROS generation. Moreover, metformin enhances the autophagy process by promoting the conversion of microtubule-associated protein 1 light chain 3 (LC3) in neuroblastoma cells (Li et al., 2019). Underexposure to Aβ, the primary cultured rat hippocampal neurons have substantial neuronal death. Metformin alleviates Aβ-induced cellular cytotoxicity and reverses hyperphosphorylation of JNK in the hippocampal neurons (Chen et al., 2016). In mouse neuroblastoma cells (Neuro-2a), the prolonged hyperinsulinemia condition induces neuronal insulin resistance and AD-associated changes, including the high level of Aβ peptide secretion and the presence of neurofibrillary tangles. Metformin can sensitize the impaired insulin actions, decrease tau phosphorylation, and inhibit NF-κB activation in mouse neurons (Gupta et al., 2011). Metformin also restores the impaired autophagy process in high glucose-cultured mouse hippocampal neuron cells (HT22), as demonstrated by increased protein levels of Beclin 1, LC3 conversion, and structure of the autophagic vacuoles. Metformin modulates autophagy through the AMPK dependent pathway (Chen et al., 2019). The protein phosphatase 2A (PP2A) appears to be the major tau phosphatase. In primary cortical neurons from C57 mice, metformin specifically reduces the tau phosphorylation at PP2A-dependent epitopes (serine 202, serine 356, and serine 262). Interestingly, metformin’s effects on PP2A activity and tau phosphorylation seem to be independent of AMPK because activation of AMPK does not influence the phosphorylation of tau at the sites analyzed. In fact, metformin can interfere with the association of the catalytic subunit of PP2A to the so-called MID1-α4 protein complex, which regulates the degradation of PP2A and thereby influences PP2A activity (Kickstein et al., 2010). The rat AD model is established by bilateral intracerebroventricular injection of streptozotocin into brains. Administration of metformin containing phosphatidylserine nanoliposomes formulation improves learning and memory of AD-rats. Metformin increases neurogenesis but significantly depresses cytokine levels of IL-1β, TNF-α, and TGF-β in rat hippocampal tissues (Saffari et al., 2020). Moreover, metformin alleviates neurodegenerative changes in streptozotocin-induced AD rats by normalization of brain glucose transport, uptake, and metabolism, paralleled with amelioration of microgliosis and astrogliosis. Metformin also preserves hippocampal synaptic plasticity in the cortical and hippocampal tissues of diabetic rats (Pilipenko et al., 2020). Metformin exerts protective effects against spatial cognitive impairment and hippocampal structure abnormalities in db/db mice. It enhances autophagic clearance in hippocampi of diabetic mice and reduces tau phosphorylation at multiple amino acid residues, such as serine 396, serine 404, and seine 202/threonine 205 (Chen et al., 2019). APP/PS1 double transgenic mice spontaneously develop AD-like cognitive deficits. Metformin attenuates spatial memory deficit, neuron loss in the hippocampus and enhances neurogenesis in APP/PS1 mice. In addition, metformin administration decreases Aβ plaque load and chronic inflammation in the hippocampus and cortex. The AD-protective functions of metformin are associated with enhanced cerebral AMPK activation. Moreover, metformin suppresses the activation of p65 NF-κB and mammalian target of rapamycin (mTOR) (Ou et al., 2018). In the aging SAMP8 mouse model that exhibits spontaneous onset of AD, metformin prevents mice’s cognitive decline by decreasing phosphorylation of tau and reducing the amyloid precursor protein-C-terminal fragment (APP-C99) in mouse brains (Farr et al., 2019). However, just like two sides of the same coin, a number of studies also indicate metformin affects amyloid-β protein precursor (Aβ-PP) metabolism, leading to Aβ generation in various cellular models (Chen et al., 2009; Picone et al., 2015; Son et al., 2016). LAN5 neuroblastoma cells cultured with metformin have increased mRNA and protein levels of Aβ-PP, concurrent with the formation of Aβ fragments and aggregates. Moreover, metformin treatment induces oxidative stress and mitochondrial dysfunction by increasing genes associated with ROS production (*NOX2*, *NOX5*, *COX1*, and *COX2*). The antioxidants ferulic acid and curcumin revert Aβ-PP levels induced by metformin (Picone et al., 2015). In mouse primary cortical neurons and N2a neuroblastoma cells stably expressing human Aβ-PP, metformin increases cellular Aβ generation. It is attributable to increased β-cleavage because metformin transcriptionally up-regulates β-secretase. Inhibition of AMPK largely suppresses metformin’s effect on Aβ generation and β-secretase transcription (Chen et al., 2009). In human neuroblastoma SH-SY5Y cells, metformin is found to enhance γ-secretase-mediated cleavage of Aβ-PP. The activated AMPK by metformin suppresses mTOR and promotes the accumulation of autophagosomes, resulting in increased γ-secretase activity and Aβ generation in cells (Son et al., 2016). C57 mice administrated with metformin exhibit activation of AMPK and increased levels of β-secretase, Aβ-PP, and aggregation of Aβ in the cortex region of mouse brains. Besides that, metformin is able to directly interact with Aβ, influencing its aggregation kinetics and features (Picone et al., 2016).

From a clinical perspective, there remains a debate in terms of metformin’s effects on AD. In a small non-diabetic cohort (*n* = 20) with mild cognitive impairment or mild dementia due to AD, metformin exposure for eight weeks is found to be safe, well-tolerated. It improves learning, memory, and attention (Koenig et al., 2017). In contrast, metformin fails to rescue the impaired cognitive performance in diabetic participants. It is even associated with worse performance (adjusted OR: 2.23, 95% CI = 1.05–4.75) than non-metformin users. Vitamin B_12_ and calcium supplements may alleviate metformin-induced cognitive impairments (Moore et al., 2013). The cohort data from National Alzheimer’s Coordinating Center suggests that the association between metformin use and better memory performance overtime is only observed in diabetic patients with normal cognition (*n* = 1192). By comparison, in patients with AD (*n* = 807), dipeptidyl peptidase-4 inhibitor treatment is associated with a slower memory decline (Wu et al., 2020). In a pooled study including five population-based cohorts (3,590 individuals with diabetes), no significant associations are found between metformin use and brain function and structure outcomes (Weinstein et al., 2019). Among 7,086 AD individuals extracted from the United Kingdom-based General Practice Research database, long-term users of metformin prescriptions are at greater risk of developing AD (adjusted OR: 1.71, 95% CI = 1.12–2.60) (Imfeld et al., 2012). In light of these clinical findings, metformin’s benefit and potential risk to patients with neurodegenerative disorders needs to be scrutinized.

## Cancer

It has been well acknowledged that inflammation is a critical component of tumor progression. Many cancers arise from sites of infection, chronic irritation, and inflammation. Moreover, the tumor microenvironment is primarily orchestrated by inflammatory cells, an indispensable participant in the neoplastic process, fostering proliferation, survival, and migration of tumor cells (Coussens and Werb, 2002). Metformin and 5-aminosalicylic acid (5-ASA) cooperate to decrease cellular proliferation and induce apoptosis of colorectal cancer cells (HCT-116 and Caco-2 cell). Metformin strengthens the anti-inflammatory effect of 5-ASA by suppressing the expression of IL-1β, IL-6, COX-2, TNF-α, and TNF receptors in cancer cells. The combination also shows metastasis-inhibitory effects via inhibiting the enzymatic activity of matrix metalloproteinase (MMP)-2 and MMP-9 (Saber et al., 2016). Metformin decreases the influx of glucose and glutamine in multiple cancer cells (HCT-116, SW480, HeLa, and MCF-7 cells) by inhibiting expressions of glucose transporter-1 and solute carrier family -1 member 5 (SLC1A5) (Ding et al., 2019). Malignant cells create an inflammatory microenvironment through releasing inflammatory cytokines and chemokines, particularly the IL-8. Metformin treatment dampens the nuclear translocation of NF-κB in LPS-treated HEK293/TLR4 cells, leading to suppressed IL-8 expression and decreased cellular migration (Xiao et al., 2017). In a transgenic zebrafish hepatocellular carcinoma model, Metformin can reduce the development of hepatocellular carcinoma by repressing diet-induced angiogenesis, steatosis, lipo-toxicity, and non-resolving inflammation. Meanwhile, metformin can restore T cell infiltration and potential surveillance (de Oliveira et al., 2019). By skewing RAW264.7 macrophages toward M2 polarization with decreased MCP-1 secretion, the metformin-treated macrophages increase apoptosis, inhibit proliferation, and decrease migration of the co-cultured HepG2 cells. Metformin pre-treatment activates Notch signaling in macrophages but represses it in HepG2 cells (Chen et al., 2015). Metformin significantly inhibits IL-8 production in human colon cancer cells (COLO205) stimulated with TNF-α, concurrent with weakened NF-κB transcriptional activity in cells. Metformin treatment inhibits colitis-associated colon tumorigenesis in C57 mice induced by azoxymethane and dextran sulfate sodium (Koh et al., 2014). The male NOD/SCIDs mice develop xenograft by inoculating HCT116 cells. The combination of rapamycin, metformin, and probiotics markedly delays tumor formation and reduces tumor size. The combination also suppresses the generation of ROS and inflammatory cytokines (IL-3, IL-6, and TNFα), associated with decreased phosphorylation of mTOR in tumors (Geagea et al., 2019). Metformin together with rapamycin attenuates the progression of prostatic intraepithelial neoplasia lesions to adenocarcinomas in the ventral prostate of HiMyc mice. The inhibitory effects of drug combination are more effective than metformin alone. The reduction of mTOR signaling by rapamycin treatment can be further potentiated by the combination with metformin, which is demonstrated by hypo-phosphorylation of mTOR at serine 2448 in the ventral prostate of mice (Saha et al., 2015). Of note, metformin is able to mimic the tumor-suppressing effects of calorie restriction (CR). As a consequence, the growth of ovarian cancer in C57 mice implanted with ID8 mouse ovarian cancer cells is hindered by treatment with metformin. The inhibitory effect of metformin is similar to treatment with a CR diet. The levels of growth factors [insulin-like growth factor-1(IGF-1), insulin, and leptin], inflammatory cytokines (MCP-1, IL-6), and vascular endothelial growth factor (VEGF) in plasma and ascitic fluid are significantly reduced by metformin. Moreover, Akt and mTOR’s phosphorylation is inhibited by metformin in mice’s peritoneal and adipose tissue (Al-Wahab et al., 2015). Swiss H mice exposed to cigarette smoke for four months, starting at birth, have preneoplastic lesions, oxidative DNA damage, and extensive downregulation of microRNAs in lung tissues. Metformin treatment prevents preneoplastic lesions, decreases DNA adduct levels and oxidative DNA damage, concurrent with the normalized expression of microRNAs (Izzotti et al., 2014).

Clinical investigations demonstrate that the tumor stroma of patients who have ovarian cancer and receive metformin treatment exhibits lower IL6 expression (Xu et al., 2018). The sera from polycystic ovary syndrome women after metformin treatment for six months exerts anti-invasive and anti-metastatic effects on human endometrial carcinoma cells *in vitro* (Tan et al., 2011). A phase II randomized trial reveals that patients with breast and colorectal cancer show a trend toward reduction of hs-CRP (−13.9%), soluble TNFα receptor-2 (−10.4%), or IL-6 (−22.9%) after metformin treatment. The study further concludes that, in non-diabetic patients with low baseline physical activity, exercise and metformin can reduce biomarkers of inflammation associated with cancer recurrence and mortality (Brown et al., 2020). Moreover, one meta-analysis indicates metformin exerts beneficial effects to reduce head and neck cancer (RR = 0.71, 95% CI = 0.61–0.84) and increase overall survival (RR = 1.71, 95% CI = 1.20–2.42). There exists a dose-response relationship and increased benefit when metformin is administered alone (Saka Herran et al., 2018). Instead, in a large cohort of 87,600 new users of metformin or sulfonylureas from the Health Improvement Network database, metformin is not found to be associated with a decreased risk of bladder cancer, and without duration-response relationship (Mamtani et al., 2014). A retrospective study comprising 1520 patients with breast cancer finds that, although metformin therapy reduces insulin, sex hormones, hs-CRP, blood glucose, and lipid profile, the overall survival is not significantly better in the metformin arm than the control arm. The progression-free survival is not different between the arms (Zhang et al., 2019).

## Conclusion

**FIGURE 1:**
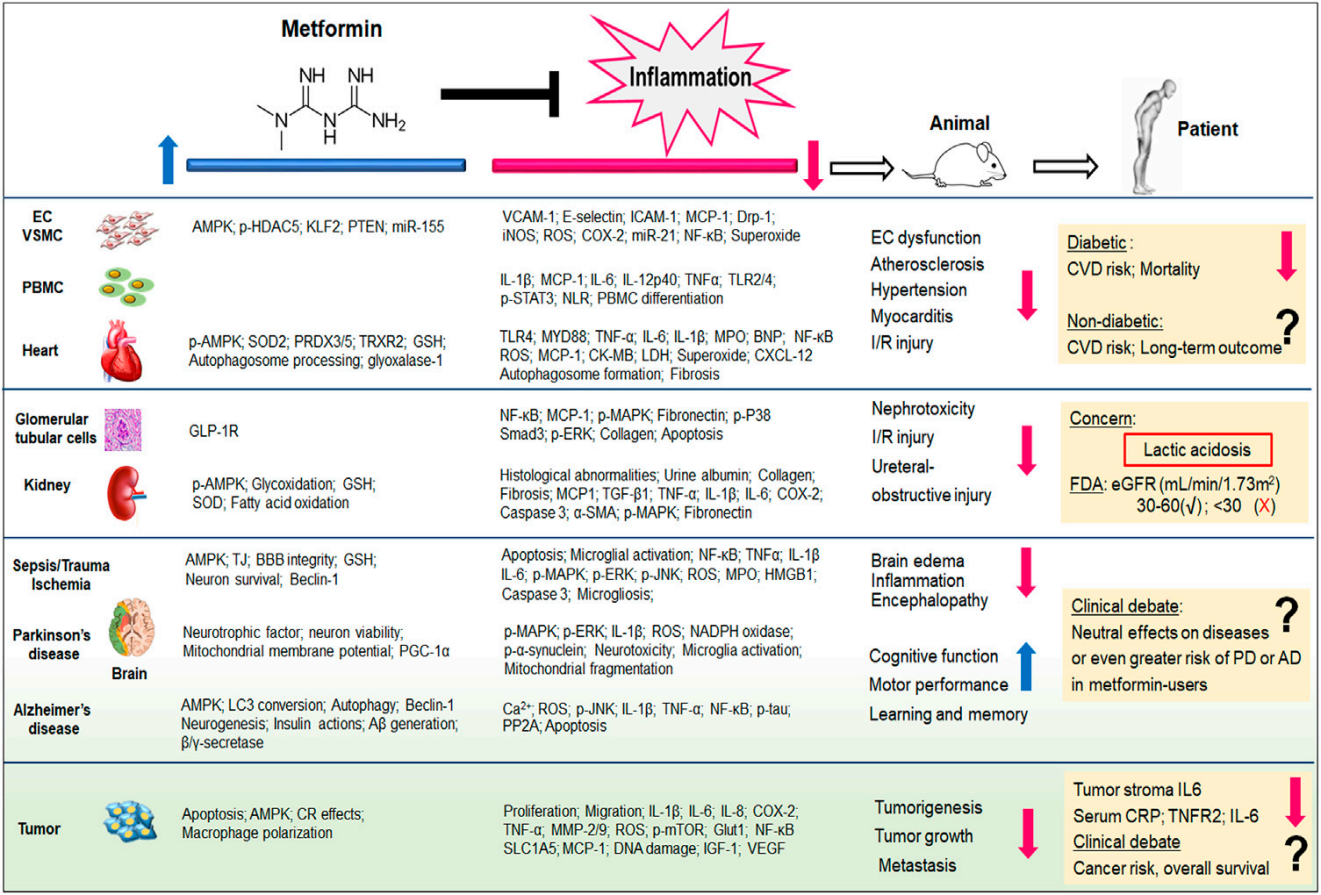
Metformin exhibits potent inflammation-inhibitory effects, irrespective of its capability of glucose control. Both pre-clinical (from cells and animal models) and clinical (from patients) evidence demonstrate the therapeutic potentials of metformin to cardiovascular diseases, kidney diseases, neurodegenerative diseases, as well as cancer. The pleiotropic actions of metformin and its anti-inflammatory properties have been reviewed in this article. **Aβ**, amyloid beta; **AD**, alzheimer’s disease; **BBB**, blood-brain barrier; **BNP**, B-type natriuretic peptide; **CK-MB**, creatinine kinase-myocardial band; **COX**, cyclooxygenase; **CR**, calorie restriction; **CRP**, C-reactive protein; **CVD**, cardiovascular diseases; **CXCL12**, C-X-C motif chemokines ligand 12; **Drp-1**, dynamin-related protein-1; **EC**, endothelial cell; **ERK**, extracellular-signal regulated kinases; **eGFR**, estimated glomerular filtration rate; **FDA**, Food and Drug Administration; **GLP-1R**, glucagon-like peptide-1 receptor; **Glut1**, Glucose transporter 1; **GSH**, glutathione; **HDAC5**, histone deacetylase 5; **HMGB1**, high mobility group box; **ICAM-1**, intercellular adhesion molecule-1; **IL**, interleukin; **iNOS**, inducible nitric oxide synthase; **I/R**, ischemia/reperfusion; **KLF2**, kruppel-like factor 2; **MAPK**, mitogen-activated protein kinase; **LDH**, lactate dehydrogenase; **MCP-1**, monocyte chemoattractant protein-1; **miR**, microRNA; **MMP**, matrix metalloproteinase; **MPO**, myeloperoxidase; **mTOR**, mammalian target of rapamycin; **MYD88**, myeloid differentiation protein 88; **NLR**, Neutrophil-Lymphocyte ratio; **p**, phosphorylation; **PBMC**, peripheral blood mononuclear cells; **PD**, Parkinson’s disease; **PP2A**, protein phosphatase 2A; **PTEN**, phosphatase and tensin homolog; **PGF2α**, prostaglandin F2α; **PRDX**, peroxiredoxin; **ROS**, reactive oxygen species; **Smad3**, SMAD family member 3; **SLC1A5**, solute-carrier family 1 member 5; **SOD2**, superoxide dismutase 2; **TGF-β**, transforming growth factor beta; **TJ**, tight junction; **TLR**, toll-like receptor; **TNF**, tumor necrosis factor; **TRXR**, thioredoxin reductase; **STAT3**, signal transducer and activator of transcription 3; **VCAM-1**, vascular cell adhesion molecule 1; **VSMC**, vascular smooth muscle cell. The blue arrow indicates the up-regulatory effects of metformin, whereas the red arrow indicates metformin’s down-regulatory effects.

A variety of evidence from cells, animal models, patient records, and randomized clinical trials strongly suggest the anti-inflammatory effects should be considered a potentially important aspect of metformin’s pharmacology (Figure 1). First of all, inflammation has been undoubtedly recognized as an important contributor to CVD. Given that the existing nonsteroidal anti-inflammatory drugs and anti-TNF drugs have shown limited utility in CVD patients, the novel agents with different inflammation-inhibitory mechanisms are worth pursuing. The anti-inflammatory effects of metformin are evident in pre-clinical models. It is also very encouraging that clinical findings have verified the protective effects of metformin in diabetic CVD cohorts. By comparison, there are still perplexities to be addressed in repurposing metformin to emerging non-diabetic CVD treatments. Secondly, the pre-clinical studies prove that metformin exerts renal-protective effects by abating inflammatory insults. We have to recognize the controversial outcomes of metformin treatment have sparked debate regarding its therapeutic efficacy in the clinical setting of kidney diseases. The particular concern regarding the safety and efficiency of metformin derives from the risk of metformin-associated lactic acidosis. Although metformin’s application has been relaxed among patients with mild to moderate renal impairment, randomized controlled trials with larger sample sizes are still waiting to be established to further validate its renal protective properties. Thirdly, it is likely that metformin exerts pleiotropic effects by targeting different molecules in brain tissues. Therefore, it is understandable that the impact of metformin on neurodegenerative diseases is complex and dependent on the type and the nature of the neuron injuries. Despite the compelling evidence that has demonstrated metformin’s actions to antagonize neuroinflammation and oxidative stress in cells or animals, unfortunately, clinical investigations have not provided convincing evidence to consolidate its translational values to treat neurodegenerative diseases. Of note, the pooled analysis even suggests the worse consequence in patients with metformin exposure. As such, more studies are required to scrutinize metformin’s benefits and the potential risk to patients and render a better understanding of the underlying pathogenic mechanism. Similarly, the evidence from both observational and laboratory studies suggest that metformin has antineoplastic activity, in part by its capability to antagonize inflammation and modulate immunity. A careful reassessment is still warranted to figure out metformin’s utilization in cancer therapy. It would be an active field investigated in depth. In summary, metformin is a safe, inexpensive medication with a history of more than 50 years of clinical experience in treating patients with T2D. Future randomized controlled trials, incorporating better stratification/targeting, would establish metformin’s utility in other emerging clinical domains, particularly for inflammation-driven chronic diseases.

## Author Contributions

BB and HC conducted the literature review, drafted the manuscript, and prepared the figure. All authors contributed to the article and approved the submission.

## Funding

This work was supported by the Seed Funding for Young Individual Research of Shenzhen Second People’s Hospital (No. 4001019).

## Conflict of Interest

The authors declare that the research was conducted in the absence of any commercial or financial relationships that could be construed as a potential conflict of interest.
